# Local Therapy Can Enhance the Prognosis of Certain Patients with Pathologically Diagnosed Neuroendocrine Prostate Carcinoma

**DOI:** 10.3390/life15050797

**Published:** 2025-05-17

**Authors:** Shoichi Kimura, Naoki Terada, Shinnya Soumiya, Takayuki Goto, Hiromitsu Negoro, Shoichiro Mukai, Osamu Ogawa, Shusuke Akamatsu, Takashi Kobayashi, Atsuro Sawada, Toshiyuki Kamoto

**Affiliations:** 1 Department of Urology, Faculty of Medicine, University of Miyazaki, Miyazaki 889-1601, Japan; shoichi_kimura@med.miyazaki-u.ac.jp (S.K.);; 2 Department of Urology, Faculty of Medicine, University of Fukui Hospital, Fukui 910-1193, Japan; 3 Department of Urology, Kyoto University Graduate School of Medicine, Kyoto 606-8507, Japan; 4 Department of Urology, Institute of Medicine, University of Tsukuba, Ibaraki 305-8576, Japan; 5 Department of Urology, Faculty of Medicine, Nagoya University Graduate School of Medicine, Aichi 466-8550, Japan

**Keywords:** neuroendocrine prostate carcinoma, local therapy, prostate cancer

## Abstract

Neuroendocrine prostate cancer (NEPC) has a poor prognosis. We performed a retrospective analysis of the factors contributing to survival in patients with histologically diagnosed NEPC. Patients pathologically diagnosed with NEPC between 2007 and 2018 were retrospectively analyzed. Overall survival (OS) from the time of the initial prostate cancer diagnosis was evaluated using the Kaplan–Meier method. Cox proportional hazards analyses were performed to evaluate the association of OS with variables including the presence of metastasis, receipt of local therapy, and disease classification (primary NEPC [p-NEPC] or treatment-related NEPC [t-NEPC]). Among 32 patients (p-NEPC, 22; t-NEPC, 10), distant metastases were identified in 25 (78%) patients, and local therapies including radical prostatectomy and local radiotherapy were provided to 21 (66%) patients. In the univariate Cox proportional hazard analyses, patients who received local therapy had a significantly lower risk of death than those who did not receive local therapy (hazard ratio = 0.284, 95% confidence interval = 0.109–0.738, *p* = 0.01). OS was significantly longer for patients receiving local therapy than for those who did not receive local therapy (36 months vs. 13 months, *p* = 0.0058). Our findings suggest the potential benefit of local therapy in the treatment of NEPC.

## 1. Introduction

Worldwide, it is estimated that there are approximately 1.4 million new prostate cancer cases and 396,792 related deaths annually, with prostate cancer ranking as the second leading cause of cancer death among men and the fifth most common cause of cancer-related mortality overall in 2022 [1]. Prostate cancer has historically been recognized as a hormone-dependent malignancy, with androgen deprivation therapy (ADT) serving as the cornerstone of treatment for decades. Although the majority of patients initially respond well to ADT, many eventually progress to a castration-resistant state (CRPC), which poses a significant clinical challenge. In recent years, the therapeutic landscape for CRPC has expanded considerably with the introduction of novel hormonal agents—such as abiraterone and enzalutamide—as well as chemotherapeutic regimens including docetaxel and cabazitaxel [2,3]. These advances have led to improved outcomes for many patients; however, a subset of prostate cancers demonstrate an even more aggressive clinical course that remains refractory to conventional treatment. A particularly aggressive and poor-prognosis phenotype, known as neuroendocrine prostate cancer (NEPC), has emerged as a rare but clinically significant subtype, distinguished by its unique biological and molecular characteristics relative to conventional prostatic adenocarcinoma. NEPC can be broadly classified into two main subtypes. Primary NEPC (p-NEPC) is characterized by the presence of neuroendocrine differentiation at the time of diagnosis, while treatment-emergent NEPC (t-NEPC) develops via a lineage switch from prostatic adenocarcinoma under the selective pressure of hormone therapy [4]. The incidence of p-NEPC is reported to be less than 2%, while t-NEPC is on the rise due to the increased use of strong AR pathway inhibitors in recent years. Both NEPCs are associated with an aggressive phenotype and poor prognosis. Multiple studies have demonstrated that the majority of NEPC patients present with distant metastases at diagnosis, underscoring the aggressive nature and dismal prognosis of this variant [5,6]. Due to its rarity and rapid progression, NEPC is typically managed using treatment protocols originally designed for neuroendocrine carcinomas of the lung, with cisplatin and etoposide-based chemotherapy being the regimen of choice. Although ADT combined with chemotherapy remains the mainstay of treatment, optimal management strategies for NEPC continue to be debated. Some studies have reported that local treatments—such as prostatectomy or localized radiotherapy—do not significantly reduce hazard ratios (HRs) in the metastatic setting [7]. Conversely, several reports have evaluated the efficacy of local therapy for metastatic prostate cancer, but not NEPC. Antwi et al. reported that among newly diagnosed metastatic prostate cancer patients, radical prostatectomy was associated with a 72% reduction in the risk of cancer death. They also reported that brachytherapy was associated with a 54% lower risk of cancer death (HR = 0.46, 95% CI: 0.33–0.64) [8]. In addition, Culp et al. reported that among newly diagnosed metastatic prostate cancer patients, groups that underwent total prostatectomy or brachytherapy had significantly higher 5-year survival and disease-specific survival rates compared to groups that did not undergo surgery or radiation therapy [9]. However, there are no reports of a definite benefit of local therapy for NEPC with or without metastases. In this pilot study, we retrospectively examine the efficacy of local treatment modalities in patients with NEPC. By focusing specifically on the impact of local therapy in this aggressive and rapidly progressing disease, we aim to provide preliminary insights into its potential to improve patient outcomes. Because of the small number of cases of NEPC, even this study did not collect a sufficient number of cases, and although it is an exploratory study with a considerable limitation, we believe that it can provide valuable findings as a pilot study to be revisited in the future with a large number of cases collected.

## 2. Materials and Methods

### 2.1. Study Population

This study included 32 Japanese patients histologically diagnosed with NEPC via biopsy of local or metastatic lesions at Miyazaki University Hospital or Kyoto University Hospital between 2007 and 2018. In this study, patient information was obtained retrospectively from medical records. For patient follow-up, the date of last follow-up was used as the date of last survival when the patient was lost. This study was approved by the Institutional Review Board of Miyazaki University Hospital (approval number: O-0642). Patient background, treatment, and survival data were retrospectively obtained from medical records. The date of prostate cancer diagnosis was the day on which prostate cancer was initially diagnosed by prostate needle biopsy. Patients diagnosed with NEPC in the initial biopsy were categorized into the primary NEPC (p-NEPC) group. Patients who were initially diagnosed with adenocarcinoma that exhibited neuroendocrine differentiation during the course of treatment, resulting in a subsequent diagnosis of NEPC, were categorized into the treatment-related NEPC (t-NEPC) group. The existence of lymph node or distant metastasis was evaluated by computed tomography or bone scan at the first diagnosis. NSE and ProGRP levels were used to diagnose NEPC.

### 2.2. Statistical Analysis

The patient characteristics data at NEPC diagnosis were presented as medians and ranges or incidence rate. Because of the small sample size, each patient variable was not examined in a multivariate analysis but only in a univariate Cox proportional hazards analysis. The variables age, PSA, NSE, and ProGRP were each based on the overall median value, and the pathological diagnosis was divided into small cell carcinoma or other, with or without metastasis, with or without local therapy, with or without chemotherapy, and p-NEPC or t-NEPC for the univariate analysis. Next, overall survival (OS) was evaluated for the variables that were significant in the univariate analysis: receipt of local therapy (radical prostatectomy and local radiation therapy) and disease classification (p-NEPC vs. t-NEPC), as well as for the clinically important variable metastatic status. OS from the NEPC diagnosis was estimated using the Kaplan–Meier method and compared between the groups using the log rank test. All statistical analyses were performed with EZR (version 1.68) (Saitama Medical Center, Jichi Medical University, Saitama, Japan), which is a graphical user interface for R (The R Foundation for Statistical Computing, Vienna, Austria). More precisely, it is a modified version of the R commander designed to add statistical functions frequently used in biostatistics [10]. *p* < 0.05 denoted statistical significance.

## 3. Results

### 3.1. Patient Characteristics

In total, 32 patients were included in this study. All patient background data were at the time of NEPC diagnosis. The median age was 69.5 years (range: 46–85). The cohort included 22 (69%) patients in the p-NEPC group and 10 (31%) patients in the t-NEPC group. PSA was measured in all patients in this study, while NSE was measured in 26 patients (16 with p-NEPC and 10 with t-NEPC) and ProGRP in 20 patients (13 with p-NEPC and 7 with t-NEPC). The median serum PSA, NSE, and ProGRP levels were 6.1 ng/mL, 19.2 ng/mL, and 59.4 pg/mL, respectively. Distant metastases were identified in 25 (78%) patients (77% in the p-NEPC group and 80% in the t-NEPC group), including lymph node, bone, lung, liver, and adrenal metastases. The histological diagnosis of NEPC was obtained by local prostate biopsy in 95% of the patients in the p-NEPC group and 70% of those in the t-NEPC group. Other patients were diagnosed with metastatic lesion biopsy. Pure small cell carcinoma was found in 17 patients. Mixed NE carcinoma–adenocarcinoma was found in nine patients. Adenocarcinoma with NE differentiation was found in six patients. Androgen deprivation therapy was administered to 29 patients (19 with p-NEPC and 10 with t-NEPC). Chemotherapy was administered to 22 patients (12 with p-NEPC and 10 with t-NEPC). Radical prostatectomy was performed in three patients (two with p-NEPC and one with t-NEPC). Local radiotherapy to the prostate was performed for 19 patients (11 with p-NEPC and 7 with t-NEPC, Table 1).

### 3.2. Univariate Analysis Using the Cox Proportional Hazards Model

Univariate Cox proportional hazards analysis from the time of NEPC diagnosis showed that the receipt of local therapy was associated with a significantly reduced risk of death (HR = 0.284, 95% CI 0.109–0.738, *p* = 0.01), whereas p-NEPC conferred a markedly lower risk than t-NEPC (HR = 0.196, 95% CI 0.068–0.566, *p* = 0.003). No significant relationship with OS was observed for age, PSA, NSE, ProGRP, pathological subtype, metastatic status at NEPC diagnosis, or receipt of chemotherapy (all *p* > 0.05) (Table 2).

### 3.3. Survival Analysis Using the Kaplan–Meier Method

**Figure 1:**
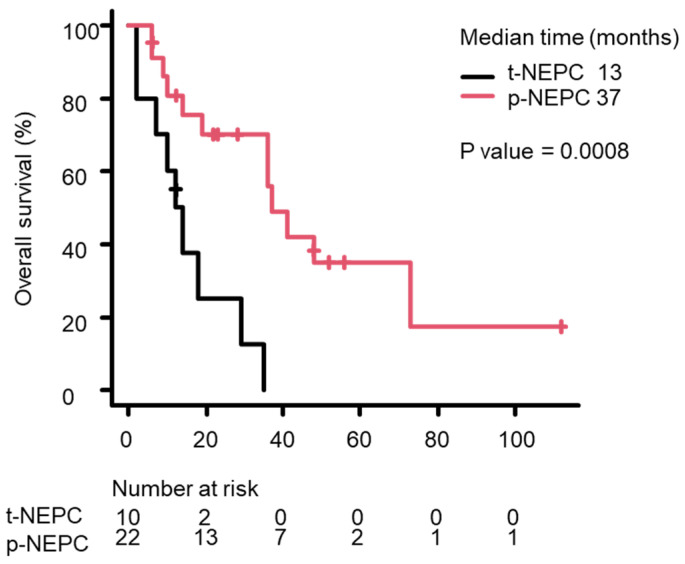
The Kaplan–Meier survival curves for OS after the NEPC diagnosis in the p-NEPC (red line) and p-NEPC (black line) groups. Median OS was 37 months in the p-NEPC group vs. 13 months in the t-NEPC group (*p* = 0.0008, log rank test).

**Figure 2:**
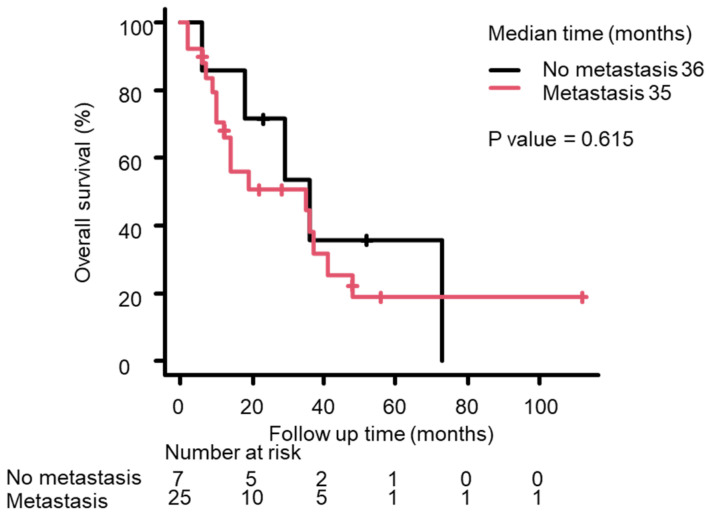
The Kaplan–Meier survival curves for OS after the NEPC diagnosis in patients with (red line) and without distant metastasis (black line) groups. Median OS was 35 months in patients with metastasis vs. 36 months in patients without metastasis (*p* = 0.615, log rank test).

**Figure 3:**
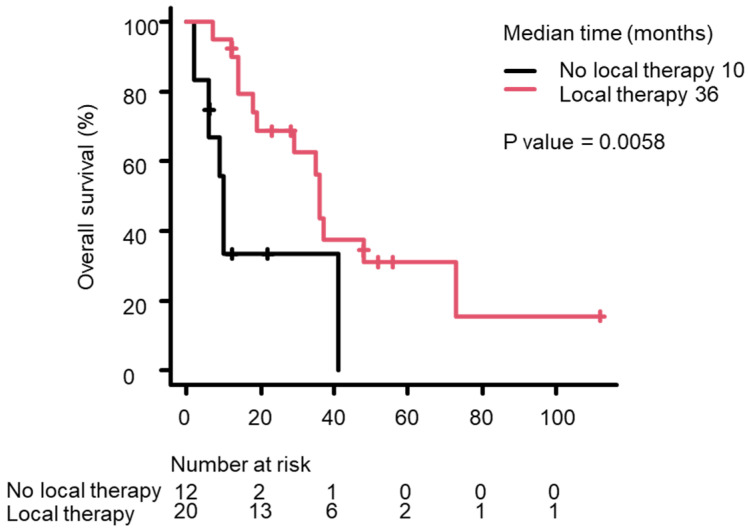
The Kaplan–Meier survival curves for OS after the NEPC diagnosis in patients who did (red line) or did not receive local therapy (black line). Median OS was 36 months in patients who received local therapy versus 10 months in patients who did not receive local therapy (*p* = 0.0007, log rank test).

Based on Kaplan–Meier survival curves, median OS from the NEPC diagnosis differed significantly between the p-NEPC and t-NEPC groups, with it being longer in the p-NEPC group than in the t-NEPC group (37 months vs. 13 months; *p* = 0.0008) (Figure 1). Median OS tended to be shorter in patients with distant metastasis at NEPC diagnosis than in those without distant metastasis, but this difference was not significant (36 months vs. 35 months; *p* = 0.615, Figure 2). Median OS was significantly longer in patients receiving local therapy—including radical prostatectomy or prostate-directed radiotherapy—than in patients who did not receive local therapy (36 months vs. 10 months; *p* = 0.0058, Figure 3). The Kaplan–Meier method allows for a simple evaluation even with a small amount of data. However, because of the small number of cases in this study, this result is not definite.

## 4. Discussion

NEPC is a rare pathological disease that comprises 0.2–1% of all prostate cancers [11,12]. According to a survey using surveillance epidemiology and end results (SEER) data, 64% of patients diagnosed with NEPC at the first visit in the United States already had metastases, and their median survival was 10 months, highlighting the extremely poor prognosis of this disease [13]. In our study, the median OS of p-NEPC was 37 months, exceeding that in previous reports. This was partially attributable to the fact that not all patients with p-NEPC had pure small cell carcinoma. In 9 patients (41%) with pure small cell carcinoma among the 22 patients with 22 p-NEPC, median OS was 26 months. We believe that this is most likely due to the success of early detection and early local therapy in the case of p-NEPC. The possibility of a large selection bias cannot be ruled out, since the number of cases in this study was small and influenced by some cases with a favorable prognosis. However, it is interesting that the results suggest that local therapy may be useful for certain patients with NEPC, and it is worthwhile conducting a larger study in the future. Neuroendocrine differentiation was reported in 17% of patients initially diagnosed with adenocarcinoma that progressed to castration-resistant prostate cancer after the initiation of ADT [5]. The frequency of neuroendocrine differentiation was correlated with the duration of ADT [14,15]. These results suggest that the NEPC is generated through shrinkage of the adenocarcinoma component, and the neuroendocrine carcinoma component remains and grows during ADT. Such patients were classified into the t-NEPC group, and they were suggested to have different characteristics from those in the p-NEPC group. In our study, OS from the initial diagnosis of prostate cancer was not significantly different between the p-NEPC and t-NEPC groups. However, OS from the date of NEPC diagnosis of t-NEPC was 12 months, which was significantly shorter than that for p-NEPC (*p* = 0.009). This may be due to the fact that t-NEPC patients are already being treated for prostate cancer, and if PSA is low and the patient has no symptoms, imaging studies, NSE, ProGRP evaluation, and prostate rebiopsy may be delayed during the course of the disease. The serum levels of biomarkers such as NSE and ProGRP were increased in patients with t-NEPC, although the PSA levels were not increased. The development of novel biomarkers is needed for the early diagnosis of p-NEPC [6]. Because many patients with NEPC have metastasis at the time of diagnosis, chemotherapy featuring cisplatin plus etoposide was commonly administered with or without ADT. The efficacy of chemotherapy is transient [10,16,17]. ADT was performed in 91% of cases in this study, and further chemotherapy did not reduce HR (HR: 2.435, *p*-value = 0.089). Recently, local radiotherapy was reported to be effective for low-volume metastatic prostate cancer in prospective randomized studies [18,19]. Local radiation therapy for metastatic prostate cancer has also been reported to significantly improve OS in Japanese patients [20,21]. However, this study did not examine the absence of metastases or whether the metastatic volume is low or high, so the benefit of receiving local therapy based on the presence of metastases needs to be evaluated in the future. Previous reports using the national cancer database suggested that local radiotherapy and radical prostatectomy might improve the prognosis of NEPC without metastasis [22]. In this study, it is possible that there is a strong bias that local therapy was successful in some cases and resulted in a long-term prognosis. In the future, it will be necessary to accumulate more cases to evaluate which patients would benefit from local therapy. The number of cases was too small to perform a multivariate analysis. Furthermore, the Kaplan–Meier analysis was affected by a large bias. Therefore, we would like to accumulate more cases and conduct a large-scale study in the future.

## 5. Conclusions

Although this study was retrospective and limited by a small number of cases, we suggest the potential of local therapy to improve the prognosis of NEPC. In the future, we would like to accumulate cases to determine which NEPC patients would benefit from local therapy.

## Figures and Tables

**Table 1:** Patient characteristics at NEPC diagnosis (*n* = 32).

Patient Characteristics	Median (Lowest–Highest) or Number (Percentage)
Median age (year)	68 (46–85)
Median PSA level (ng/mL)	6.1 (0.01–325)
Median NSE level (ng/mL)	19.2 (7.3–268.3)
Median ProGRP level (pg/mL)	59.4 (34.1–829)
[Primary (p) or treatment-related (t) NEPC]	
p-NEPC	22 (69%)
t-NEPC	10 (31%)
[Pathological diagnosis]	
Small cell carcinoma	17 (53%)
Mixed NE carcinoma–adenocarcinoma	9 (28%)
Adenocarinoma with NE differentiation	6 (19%)
[Metastatic lesion]	
Any	25 (78%)
Lymph node	21 (66%)
Bone	19 (59%)
Lung	5 (16%)
Liver	3 (9%)
Adrenal	1 (3%)
[Treatment]	
Radical prostatectomy	3 (9%)
Local radiotherapy	18 (56%)
Androgen deprivation therapy	29 (91%)
Chemotherapy	22 (69%)

**Table 2:** Univariate Cox proportional hazard analysis of patient factors for OS.

	*n*	HR (95% CI)	*p*-Value
Age			
≦69.5	16		
>69.5	16	1.236 (0.522–2.93)	0.623
PSA			
≦6.1	16		
>6.1	16	0.8179 (0.338–1.98)	0.66
NSE			
≦19.2	13		
>19.2	13	1.138 (0.954–1.357)	0.15
ProGRP			
≦59.1	10		
>59.1	10	3.402 (0.835–13.86)	0.087
Pathological diagnosis			
Mixed NE carcinoma–adenocarcinoma/ Adenocarinoma with NE differentiation	15		
Small cell carcinoma	17	1.648 (0.691–3.928)	0.26
Metastasis/no metastasis	25/7	1.292 (0.469–3.559)	0.62
Local therapy/no local therapy	21/11	0.284 (0.109–0.738)	0.01 *
Chemotherapy/no chemotherapy	22/10	2.435 (0.873–6.795)	0.089
pNEPC/t-NEPC	22/12	0.196 (0.068–0.566)	0.003 *

* *p* < 0.05.

## Data Availability

The data and materials utilized in this study are available upon request, with potential restrictions on the accessibility of specific sensitive or confidential information. For inquiries concerning access to the data and materials, kindly contact Shoichi Kimura, affiliated with the Department of Urology, Faculty of Medicine, at the University of Miyazaki.

## Abbreviations

The following abbreviations are used in this manuscript:

NEPC	Neuroendocrine prostate cancer
p-NEPC	Primary neuroendocrine prostate cancer
t-NEPC	Treatment-related neuroendocrine prostate cancer
OS	Overall survival
ADT	Androgen deprivation therapy
HRs	Hazard ratios
PSA	Prostate-specific antigen
ProGRP	Pro-gastrin-releasing peptide
NSE	Neuron-specific enolase
CI	Confidence interval
SEER	Surveillance epidemiology and end results
